# The Role of Estrogen Signaling in a Mouse Model of Inflammatory Bowel Disease: A Helicobacter Hepaticus Model

**DOI:** 10.1371/journal.pone.0094209

**Published:** 2014-04-07

**Authors:** Lydia C. Cook, Andrew E. Hillhouse, Matthew H. Myles, Dennis B. Lubahn, Elizabeth C. Bryda, J. Wade Davis, Craig L. Franklin

**Affiliations:** 1 Department of Veterinary Pathobiology, University of Missouri, Columbia, Missouri, United States of America; 2 Department of Molecular Microbiology & Immunology, University of Missouri, Columbia, Missouri, United States of America; 3 IDEXX Laboratories, Columbia, Missouri, United States of America; 4 Department of Biochemistry, University of Missouri, Columbia, Missouri, United States of America; 5 Departments of Health Management and Informatics, and Statistics, University of Missouri, Columbia, Missouri, United States of America; CWRU/UH Digestive Health Institute, United States of America

## Abstract

The pathogenesis of inflammatory bowel diseases (IBD), Crohn's disease and ulcerative colitis, is due in part to interactions between the immune system, genetics, the environment, and endogenous microbiota. Gonadal sex hormones (GSH), such as estrogen, are thought to be involved in the development of IBD as variations in disease severity occur during pregnancy, menopause, or oral contraceptives use. In certain strains of mice, infection with *Helicobacter hepaticus* triggers IBD-like mucosal inflammation that is more severe in female mice than in males, suggesting a role for GSH in this model. To determine the role of estrogen signaling in microbiota-induced intestinal inflammation, estrogen receptor (ER) α and β knock-out (KO) mice, ER agonists, and adoptive transfers were utilized. We demonstrate that, when signaling is limited to ERβ on a non-CD4^+^ cell subset, disease is less severe and this correlates with decreased expression of pro-inflammatory mediators.

## Introduction

Crohn's disease (CD) and ulcerative colitis (UC) are classified as inflammatory bowel diseases (IBDs) and are diagnosed primarily in North America and northern Europe [1]. There are approximately one million people in the U.S. living with IBDs [2], and while the cause is unknown, differences in intestinal microbiota, hygiene, genetics, geography, and the environment can contribute to disease development [1]. There is no known cure for IBDs and current treatments are predominantly pharmaceutical, merely suppressing clinical symptoms. When pharmaceutical intervention fails, the final option for treatment is bowel resection.

ERα and ERβ modulate gene expression by directly interacting with estrogen response elements or other transcription factors [3], and have been implicated in the modulation of several immune-mediated diseases [4]. For example, physiologic events that result in elevated estrogen levels are associated with increased disease severity in systemic lupus erythematous (SLE) patients, but decreased disease severity in rheumatoid arthritis (RA) and multiple sclerosis (MS) patients [5]–[7]. Furthermore, there are distinct sex biases in colon cancer [8] and inflammatory intestinal disorders such as microscopic colitides [9], [10], suggesting a role for gonadal sex hormones (GSHs), including estrogen, in the modulation of intestinal health. GSH-related events, such as menopause, pregnancy and the use of oral contraceptives can modulate IBD. In particular, the development of IBD has been associated with oral contraceptive use [11], [12] and the cessation of oral contraceptive use has been associated with the resolution of clinical signs and symptoms [13], [14]. Furthermore, women are partially protected from disease flare-ups during pregnancy [15] and estrogen-based hormone replacement therapies are associated with decreases in disease severity in post-menopausal patients [16].

Animal models of IBD also support the use of estrogen therapies to modulate disease. For example, in an IBD model utilizing the HLA-B27 transgenic rat, estrogen administration decreases diarrhea and intestinal inflammation [17]. Further work with this model has demonstrated decreased disease severity when NF-κB signaling is inhibited by ERB-041, a selective ERβ antagonist [18]–[20]. Furthermore, estrogen administration decreases disease in other IBD models such as dinitrobenzene sulfonic acid (DNB)-induced and acetic acid-induced colitides [21], [22]. Finally, estrogen signaling has also been shown to protect against the development of colitis-associated diseases, such as neoplasia, in the azoxymethane/dextrane sodium sulfate (DSS)-induced colorectal cancer model [23].

In the *Helicobacter hepaticus-*induced model of IBD, susceptible mouse strains, such as A/J, develop histologically observable disease in the cecum three months post-inoculation [24]. While the exact mechanism by which *H. hepaticus* initiates disease is unknown, it is believed that *H. hepaticus* colonizes the cecum and serves as a provocateur for an inflammatory response against endogenous microbiota [25]–[28]. *H. hepaticus-*induced typhlitis is characterized by lymphocytic infiltration of the lamina propria and increased expression of the T_H_1 cytokine genes IL-12/23 p40 and IFN-γ, as well as TNF-α and the chemokines CXCL9 and CXCL10 [24], [29]–[31]. *H. hepaticus*-infected female A/J mice develop more severe intestinal inflammation and have significantly higher T_H_1 cytokine gene expression than infected males [29]. These data and the previously reported associations between estrogen and IBD led to the hypothesis that estrogen signaling modulates the development of *H. hepaticus-*induced inflammation. To test this hypothesis, *H. hepaticus*-infected ERα- and ERβ-knockout mice and mice treated with ER specific agonists were used. Additionally, an adoptive transfer model was used to identify the cell type through which estrogen-mediated events occur.

## Materials and Methods

### Ethics Statement

This study was conducted in accordance with the *Guide for the Care and Use of Laboratory Animals* and approved by the University of Missouri Animal Care and Use Committee [ACUC Protocol Number: 7749]. All surgical procedures were performed under isoflurane anesthesia and all mice received post-operative buprenorphine for analgesia.

### Generation of estrogen receptor (ER) and RAG2 deficient A/J Mice

Wild type A/JCr mice were obtained from Frederick National Laboratory for Cancer Research. B6;129-*Esr1^tmUNC^* (ERα knockout) [32] and B6;129-*Esr2^tmUNC^* (ERβ knockout) [33] mice were kindly provided by Dr. Dennis Lubahn of the University of Missouri. A marker-assisted congenic breeding scheme was used to transfer the ER mutations to the A/J mouse strain. Resulting strains (A.129(P2)-*Esr1^tmKsk^/Mmmh* and A.129P2(B6)-*Esr2^tmUNC^/Mmmh*) strain were subsequently submitted to the Mutant Mouse Regional Resource Center (MMRRC 029880 and 029908 respectively). C57BL/6 mice containing a disruption in the *Rag2* (recombination activating gene 2) gene were obtained from Taconic laboratories (B6.129S6-Rag2^tm1Fwa^). Again, a marker-assisted congenic breeding scheme was used to generate *Rag2*-deficient mice on an A/JCr background (A.129S6(B6)-Rag2^tm1Fwa^). The generation of mice with mutations in both *Rag2* and either ERα or ERβ was accomplished by crossing the A.129S6(B6)-Rag2^tm1Fwa^ strain with either A.129(P2)-*Esr1^tmKsk^/Mmmh* or A.129P2(B6)-*Esr2^tmUNC^/Mmmh* mice.

### ER alpha, ER beta, and RAG2 PCR

All knock-out mice used in experiments or as donors for adoptive transfers were genotyped at 3–4 weeks of age. At this time, all mice were ear-tagged (National Band & Tag, Newport, KY) and 3–5 millimeter tail snips were collected for genotyping. DNA was extracted using a Qiagen DNeasy DNA extraction kit (Valencia, CA) per manufacturer's protocol. The resulting DNA was used in PCR reactions to determine each individual mouse's genotype. Each reaction contained 0.2 μl FastStart *Taq* (5 U/μl) (Roche, Indianapolis, IN), 0.3 μl of each 25 μM primer (IDT Coralville, IA), 3.2 μl of 1.25 mM dNTPs (Roche) and 2 μl 10× PCR buffer with MgCl_2_ (Roche). Thermal cycling conditions were: 1 cycle at 94°C for 4 min, 45 cycles of 15 at 94°C, 45 sec at the appropriate annealing temperature (*Table 1*), 1 min at 72°C and 1 cycle at 72°C for 10 min. PCR reactions were analyzed by gel electrophoresis with 3% 1× TBE agarose gels and amplicons were visualized with ethidium bromide (Bio-rad Hercules, CA). Primer sequences and annealing temperatures are listed in *Table 1*.

**Table 1:** Genotyping PCR Primers, Products, & Annealing Temperatures.

PCR Primers	Forward (5′-3′)	Reverse (5′-3′)	Fragment size (bp)	Annealing Temp (°C)
**ER α WT**	TCTACGGCCAGTCGGGCATC	TAGGCGACACGCTGTTGAGCTG	102	66
**ER β WT**	TGACAATGGCTGTGTTCTCTGCTC	TTAAAGTTAAACGCCAGCCCACCG	566	66
**neo cassette**	5′-TGCCTGCTCTTTACTGAAGGCTCT-3′		α – 363 β - 430	66
**RAG2 WT**	GGGAGGACACTCACTTGCCAGTA	AGTCAGGAGTCTCCATCTCACTGA	263	62
**neo cassette**	5′-CGGCCGGAGAACCTGCGTGCAA-3′		350	62

### Bacterial Culture

Culture of *Helicobacter hepaticus* strain MU-94 was previously described [24], [29]. Mice were inoculated via gastric gavage with 0.5 ml of culture containing approximately 5×10^8^ bacteria/mL. Colonization was confirmed 2–4 weeks post-inoculation and at necropsy with *H. hepaticus-*specific PCR of feces [34].

### Ovariectomy and Pellet Placement

Mice were anesthetized with isoflurane, clipped dorsally, and aseptically scrubbed. A single longitudinal skin incision was made on the dorsal midline. The ovaries were visualized, the muscle wall was incised, and ovaries were removed. After a continuous-release hormone pellet (described below) was inserted rostrally into the subcutaneous space between the scapulae through the initial incision, the incision was closed with sterile skin staples. Mice were given 0.0015 mg buprenorphine IP for analgesia and recovered on a heating pad. Seven to ten days post-operatively, the skin staples were removed. At the time of necropsy, mice with detectable ovarian remnants were removed from the study.

### Compounds

In initial studies, a 90-day continuous-release hormone pellet containing 1.5 mg/pellet of 17β-estradiol (E2) or a placebo (Innovative Research of America, Sarasota, FL) was administered. The secondary studies utilized 90-day continuous-release pellets containing either 2.5 mg of the ERα agonist, PPT (4,4′,4″-(4-Propyl-[1H]-pyrazole-1,3,5-triyl) trisphenol), or 10 mg of the ERβ agonist, DPN (diarylpropionitrile 2,3-bis(4-hydroxyphenyl)-propionitrile) (Tocris Bioscience Ellisville, MO and Innovative Research of America). At the time of necropsy, the presence of a pellet was confirmed and mice without a visible, intact pellet were removed from the study.

### Isolation of CD4^+^ Lymphocytes and Adoptive Transfers

CD4^+^ T cells were isolated from the spleens of 8–12 week old donor mice. CD4^+^ T cells were negatively selected using a CD4^+^ T Cell Isolation Kit (Miltenyi Biotec, Auburn, CA), and purified with a Miltenyi Biotec autoMACS cell separator. Purity of the CD4^+^ cells was accessed following isolation. This was accomplished by staining cells with a FITC-conjugated CD4 (eBioscience, San Diego, CA); following staining, cells were washed, suspended in an appropriate volume of staining buffer and analyzed with either a FACScan or FACSCalibur flow cytometer (BD Biosciences). All adoptively transferred cell populations were >83% pure for CD4+ expression (average purity  = 88%). After isolation, cells were suspended in sterile PBS and 5×10^5^ cells were injected into the tail vein of recipient mice. All mice were monitored for weight loss; loss of more than 20% of the starting body weight resulted in removal from the study.

### Necropsy

In all experiments, the cecum was removed and cecal contents collected to confirm *H. hepaticus* colonization by PCR. The cecum was then sectioned longitudinally into two equal parts. One half was rinsed with sterile PBS and flash frozen in liquid nitrogen for use in mRNA expression analyses. The other half was fixed in zinc fixative and embedded in paraffin for histologic evaluation.

### Histological Evaluation

Cecal histologic sections were stained with hematoxylin and eosin (H&E) stain and scored for inflammatory lesions using a previously described protocol [29], [31], [35]. In short, a longitudinal cecal section from the ileocecal junction to the cecal tip was scored in a blinded fashion. The cecum as a whole was assigned a category score for each of the following: inflammation intensity (0 =  none, 1 =  mild, 2 =  moderate, 3 =  severe); longitudinal extent of inflammation (0 =  none, 1 = 1–2 small foci, 2 =  patchy, 3 =  diffuse); vertical extent of inflammation (0 =  none, 1 =  basal mucosal inflammation, 2 =  full thickness mucosal inflammation, 3 =  transmural inflammation); and hyperplasia (0 =  none, 1 =  focal, 2 =  patchy hyperplasia, 3 =  diffuse hyperplasia). The lesions scores depicted in figures are the sum of the four category scores; any lesions score greater than 2 were decreased by 2 to minimize inflation of scores by inflammation alone, which is described by three of the four categories evaluated. Lesion scores in both the knockout and agonist treatment studies were found to have a bimodal distribution with a score of 6 being at the center of this distribution. As a result, differences in severe disease (scores greater than or equal to 6) incidence between test groups and control groups were also examined.

### RNA Isolation and Reverse Transcription

Frozen cecal sections were thawed in TRIzol (Invitrogen, Carlsbad, CA) and homogenized using a Tissuelyser (Qiagen, Valencia, CA). Total RNA was isolated per manufacturer's (Invitrogen) instructions. Five micrograms of total RNA was reverse-transcribed using the Super Script II kit (Invitrogen, Carlsbad, CA) utilizing the manufacturer's oligo(dT) primer protocol. The resultant cDNA was diluted to a final concentration of 20 ng/μL.

### Real-Time PCR

Real-time quantitative PCR was performed either with a Roche Light Cycler 2.0 or a Qiagen Rotor-Gene Q using Qiagen Quanti-tect SYBR Green (Qiagen, Valencia, CA). The run conditions used for real-time PCR have been previously described [24] and primers can be found in *Table 2* *.*

**Table 2:** Real time PCR Primers and Annealing Temperatures.

PCR Primers	Forward (5′-3′)	Reverse (5′-3′)	Annealing Temp (°C)
**HPRT**	GTAATGATCAGTCAACGGGGGAC	CCAGCAAGCTTGCAACCTTAACCA	60
**CXCL9**	ACTCGGCAAATGTGAAGAAG	CCCATTAAGATTCAGGGTGC	58
**IFN-γ**	GCCATCAGCAACAACATAAGC	TGGGACAATCTCTTCCCCAC	58
**IL-4**	ATGGGTCTCAACCCCCAGCTAGT	GCTCTTTAGGCTTTCCAGGAAGTC	57
**IL-10**	CGGGAAGACAATAACTG	CATTTCCGATAAGGCTTGG	58
**IL-12/23 p40**	ACTCACATCTGCTGCTCCAC	GGGAACTGCTACTGCTCTTGA	60
**IL-17a**	CAGAAGGCCCTCAGACTACC	CTTTCCCTCCGCATTGACAC	56.6
**IL-17f**	ACTTGCCATTCTGAGGGAGG	TGATGCAGCCTGAGTGTCTG	57.1
**IL-23 p19**	CCAGCGGGACATATGAATCTAC	GATGTCAGAGTCAAGCAGGTG	55.3

### Statistical Analysis

One-way ANOVAs with Student Newman-Keuls post-hoc tests were used for analysis of lesion scores that passed (p<0.05) a goodness-of-fit test for normality (Shapiro-Wilk) performed using SigmaPlot 11.0 (Systat Software Inc., San Jose, CA). For lesions scores that were not normally distributed, either the exact Wilcoxon-Mann-Whitney test (two groups) or exact Kruskal-Wallis test (three or more groups) was performed using SAS 9.3 (SAS, Cary, NC), followed by Dunn's post-hoc multiple comparisons [36]. Because evaluation of mRNA expression data showed that most of measures departed from normality, either the exact Wilcoxon-Mann-Whitney test (two groups) or exact Kruskal-Wallis test (three or more groups) was performed using SAS 9.3 (SAS, Cary, NC), followed by Dunn's post-hoc multiple comparisons. The reported p-value for the post-hoc tests are after adjustment, so that comparing *p* to α = 0.05 still preserves the family-wise error rate at 5%. Exact Chi-squared tests of independence were performed to evaluate differences in severe disease incidence. Correlations between cytokine mRNA expression and disease severity were evaluated with Spearman's correlation coefficients and corresponding p-values were adjusted by a false discovery rate (FDR) controlling method, due the large number of correlations tested. For all analyses, p-values ≤.05 (after any adjustments) were regarded as significant. For experiments involving analysis of knockout mice, preliminary studies did not detect any differences between mean lesion scores of wild type, *ERα^+/−^*, and *ERβ^+/−^* mice (mean p = .61, minimum p>.35). These groups were therefore combined as controls. All graphs and dot plots were generated using GraphPad Prism 5.0 software (GraphPad Prism Software Inc., La Jolla, CA).

## Results

### Estrogen administration decreases disease severity

To evaluate the role of estrogen, female mice were ovariectomized at 3–4 weeks of age and implanted with either a placebo (control) or continuous-release hormone pellet (1.5 mg - supraphysiologic dose of 17β-estradiol, E2). All mice were orally inoculated with *H. hepaticus* one week post-operatively. Ninety days post-inoculation, mice were sacrificed and ceca collected for the evaluation of histologic disease and mRNA expression.

**Figure 1:**
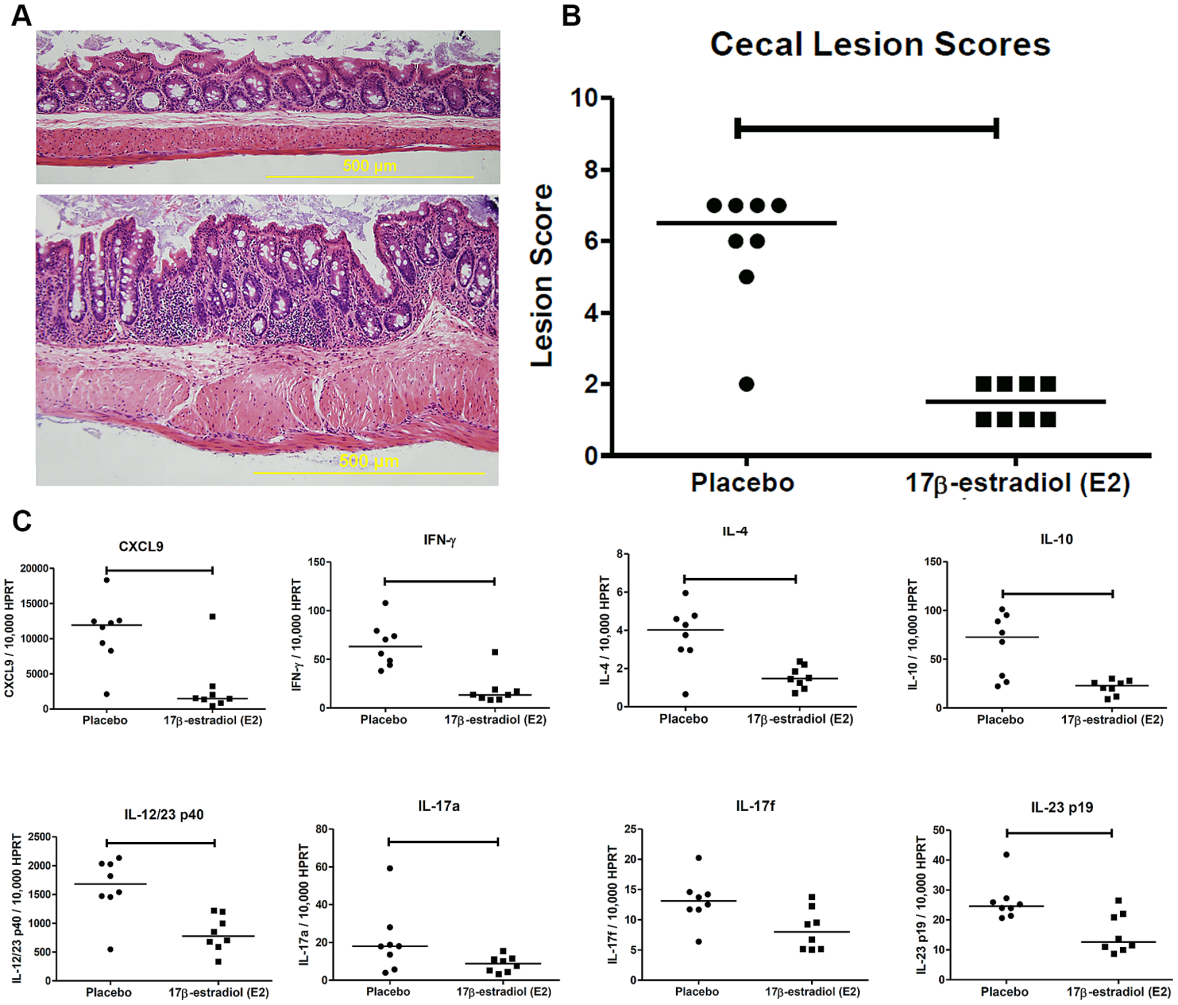
Estrogen treatment decreases disease severity and the expression of pro-inflammatory cytokines and chemokines. (A) Representative histologic images of ceca from ovariectomized A/J mice sacrificed 90 days after *Helicobacter hepaticus* inoculation and implantation with a subcutaneous pellet containing either (top) 17β-estradiol (1.5 mg/pellet) or (bottom) placebo. (B) Cecal lesion scores of placebo (circles) and estradiol (squares) treated mice (n = 8, 8 respectively). Each point represents the lesion score for an individual mouse. Lesion score data analyzed with an exact Wilcoxon-Mann-Whitney test; horizontal lines represent medians. (C) Real-time quantitative PCR measurements of cecal cytokine and chemokine mRNA expression levels in mice receiving placebo (circle) or estrogen (square) treatment (n = 8). mRNA expression was normalized to the expression of the housekeeping gene HPRT. All mRNA expression data with an exact Wilcoxon Rank Sum Test; each point represents the expression level for an individual mouse and horizontal lines represent medians. For all data analysis p≤0.05 considered significant and indicated by horizontal capped bars.

The ceca of E2-treated mice had little to no inflammatory infiltrates (Fig. 1A,top), while the placebo-treated mice had an increase in cellular infiltration and hyperplasia/dysplasia of the cecal mucosa (Fig. 1A, bottom). Evaluation of cecal histologic sections revealed that E2-treated mice had significantly lower lesion scores than those that received the placebo treatment (Fig. 1B).

We previously demonstrated that mRNA expression of IL-12/23 p40, IFN-γ and CXCL9 is elevated during *H. hepaticus-*induced intestinal inflammation [24]. Estrogen treatment significantly reduced the cecal expression of all three inflammatory mediators compared to placebo-treated mice (Fig. 1C). To evaluate the effect of estrogen treatment on other branches of the immune system expression levels of: IL-10, IL-4, IL-17a, IL-17f and IL-23 p19 were evaluated. All but IL-17f were significantly decreased with estrogen treatment. When the relationship between disease severity and cytokine expression was evaluated, positive correlations were found for *CXCL9, IL-12/23 p40*, *IFN-γ* and *IL-10* (Table S1). The overall decrease in inflammation and inflammatory gene expression suggests that estrogen treatment is capable of reducing disease severity.

### Disease severity decreases when estrogen signaling is limited to ERβ

While estrogen administration was shown to decrease disease, this finding is not physiologically or therapeutically relevant as this treatment resulted in severe bone marrow depression (data not shown) and marked decreases in expression of all cytokines measured. However, these studies served as observation generating studies that stimulated the pursuit of additional studies centered on estrogen receptor signaling. To determine the role of the two estrogen receptors, ERα and ERβ, in modulating disease, female *ERα^−/−^* and *ERβ^−/−^* mice were inoculated with *H. hepaticus* at 3–4 weeks of age. To serve as controls, female heterozygotes (*ERα^+/−^* or *ERβ^+/−^*) and wild-type littermates were inoculated. As no statically significant differences were found between wild-type and heterozygotes when lesions scores and cytokine mRNA expression was compared (median p = 0.725 among Wilcoxon-Mann-Whitney tests conducted), these mice were combined and used as controls.

**Figure 2:**
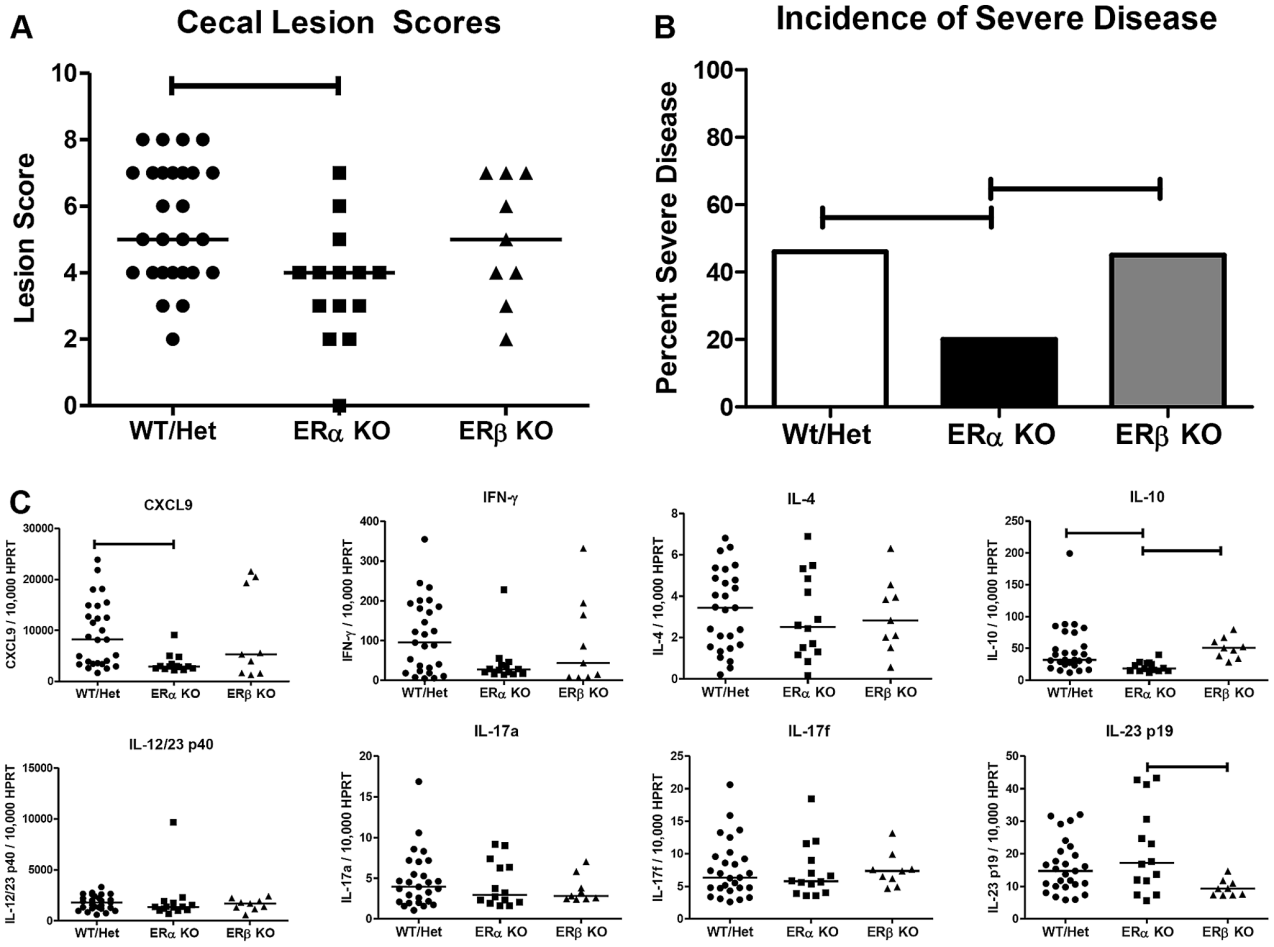
Absence of ERα expression decreases disease severity and mRNA expression of CXCL9 and IL-10. A/J female mice deficient for either ERα or ERβ expression and heterozygous/wild type littermate controls were inoculated with *H. hepaticus* and evaluated 3 months post-inoculation. (A) Cecal lesion scores of inoculated ERα (squares), ERβ (triangles) and control (circles) mice (n = 14, 9, and 27 respectively); each point represents the lesion score for an individual mouse. Lesion score data analyzed with an exact Kruskal-Wallis test followed by Dunn's post-hoc comparisons. Horizontal bars represent medians. (B) Frequency of mice with severe cecal inflammation (lesion score ≥6) within each genotype group. Data analyzed by exact Chi-squared test of independence. (C) Real-time quantitative PCR measurements of cecal cytokine and chemokine mRNA expression levels in mice deficient for ERα (squares) or ERβ (triangles) and littermate controls (circles). The mRNA expression of all cytokines were normalized to HPRT. Data analyzed with an exact Kruskal-Wallis test followed by Dunn's post-hoc comparisons; each point represents the expression level for an individual mouse and horizontal lines represent medians. For all data analysis p≤0.05 considered significant and indicated by horizontal capped bars.

Ninety days post-inoculation, mice were necropsied and their ceca assessed for histologic inflammation and mRNA expression. Lesion scores of *ERβ-*deficient mice did not differ from those of the control mice, however ERα deficiency resulted in a significant decrease in disease (Fig. 2A). *ERα*-deficient mice also had a significantly lower incidence of severe disease (lesion score ≥6) when compared to wild-type control and *ERβ*-deficient mouse groups (Fig. 2B). Associated with this decrease in disease severity and incidence of severe disease were significant decreases in expression of *CXCL9* and a trend towards decreased *IFN-γ* expression (p = 0.132). Additionally, there were positive correlations between the expression of *CXCL9, IL-12/23 p40* and *IFN-γ* and lesion scores (Table S2).

### Selective ERβ signaling decreases disease severity

The above data suggested that lack of functional ERα decreases disease development. Plausible explanations for this finding include 1) signaling through ERα is pro-inflammatory and removal of this signaling dampens disease or 2) preferential signaling through the remaining functional receptor, ERβ, dampens disease. To further assess potential modulation of disease by estrogen receptor signaling, ER-specific agonists were utilized.

Wild type A/J mice were ovariectomized at 3–4 weeks of age, implanted with an ERβ agonist (DPN), ERα agonist (PPT), or placebo continuous-release pellet, and then inoculated with *H. hepaticus* one week post-operatively. Ninety days post-inoculation, mice were sacrificed and ceca collected for evaluation.

**Figure 3:**
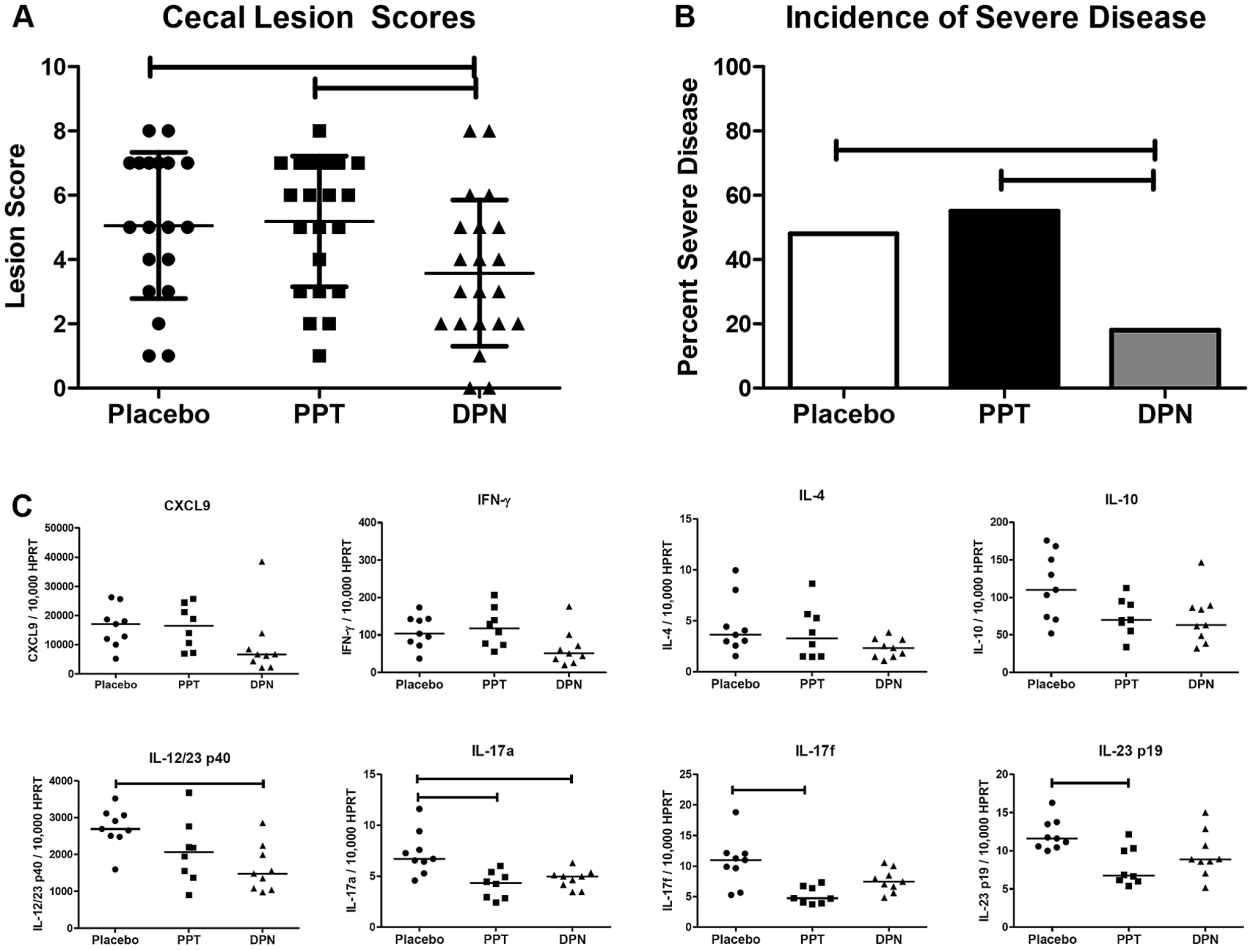
Treatment with an ERβ agonist decreases disease severity and the expression of pro-inflammatory cytokines. Female A/J mice were ovariectomized, administered continuous-release pellets containing a placebo, ERα agonist (PPT) or ERβ agonist (DPN), inoculated with *H. hepaticus*, and necropsied three months later. (A) Cecal lesion scores of mice treated with a placebo (circles), PPT (squares) or DPN (triangles) pellet (n = 19, 22, and 21 respectively). Points represent the lesion scores of individual mice. Data analyzed by one-way ANOVA with a Student-Newman-Keuls post-hoc test; data presented as group means ± s.d (B) Frequency of mice with severe disease (lesion score ≥6). Data analyzed with chi-squared analysis. (C) Real-time quantitative PCR measurements of cecal mRNA expression levels in mice treated with an ERα agonist (squares), ERβ agonist (triangles), or placebo control (circles) (n = 8, 9, and 9 respectively). The mRNA expression levels of all cytokines are normalized to HPRT expression. Data analyzed with an exact Kruskal-Wallis test followed by Dunn's post-hoc comparisons; each point represents the expression level for an individual mouse and horizontal lines represent medians. For all data analysis p≤0.05 considered significant and indicated by horizontal capped bars.

Histologic evaluation demonstrated that treatment with the ERβ agonist (DPN) was associated with significantly lower lesion scores compared to both the control and PTT treated mice (Fig. 3A), while treatment with the ERα agonist (PPT) did not alter intestinal inflammation compared to control mice. The incidence of severe disease (lesion score ≥6) in the group treated with DPN was also significantly less than both the control group and the PTT treated mice (Fig. 3B). Associated with this decreased disease severity were decreases in cecal expression of *IL-12/23 p40* and *IL-17a* as compared to placebo-treated controls. When DPN-treated mice were compared to the control and PPT-treated mouse groups there was also a trend towards decreased *IFN-γ* (p = 0.108, 0.060 respectively) and *CXCL9* (p = 0.100, 0.084 respectively) (Fig. 3C). Moreover, there were positive correlations between expression of *CXCL9* and *IFN-γ* and lesion scores (Table S3). Collectively, these results suggest that signaling through ERβ decreases disease severity and this decrease is associated with decreased expression of pro-inflammatory cytokines.

### Estrogen signaling on CD4^+^ lymphocytes does not affect disease severity

Previous studies in our laboratory have demonstrated that *H. hepaticus* induces a T_H_1 inflammatory response, suggesting a role for CD4^+^ T cells in disease development. To investigate the influence of estrogen signaling through ERα and ERβ on CD4^+^ cell populations, CD4^+^ cells with ablated ER expression were utilized in adoptive transfer experiments.

**Figure 4:**
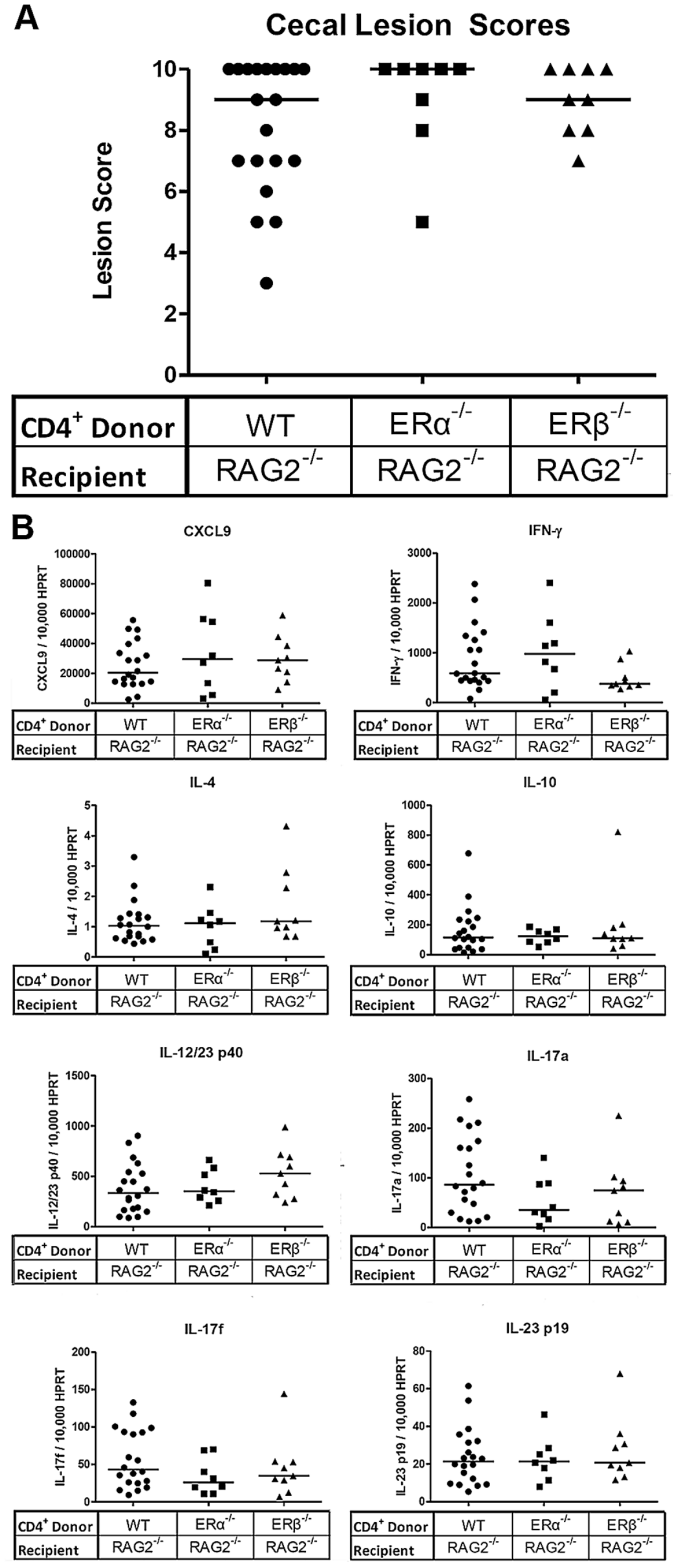
Lack of ER signaling in CD4^+^ cellular populations does not alter cecal inflammation or cytokine and chemokine expression. (A) Cecal lesion scores in female *H. hepaticus-*inoculated *RAG2* ^−/−^ mice adoptively transferred *ERα* ^−/−^ (squares) or *ERβ* ^−/−^ (triangles) CD4^+^ lymphocytes (n = 8, 9 respectively). As a control group, *RAG* ^−/−^ female mice were adoptively transferred wild type CD4^+^ lymphocytes (n = 20)(circles). Points represent the lesion scores of individual mice in each experimental group and horizontal bars indicate median lesion scores; horizontal lines represent medians. (B) Real-time quantitative PCR measurements of cecal cytokine and chemokine mRNA expression levels in *RAG2* ^−/−^ mice adoptively transferred CD4^+^ lymphocytes deficient for ERα or ERβ expression. The mRNA expression levels of all cytokines were normalized to HPRT mRNA expression. Each point represents the normalized expression of an individual mouse (symbols the same as lesion score data). All data analyzed by an exact Kruskal-Wallis test and horizontal lines represent medians. For all data analysis p≤0.05 considered significant.

Splenic CD4^+^ lymphocytes were isolated from mice deficient for either *ERα* or *ERβ* and adoptively transferred to *H. hepaticus*-inoculated *RAG2* ^−/−^ A/J mice. As a control, CD4^+^ lymphocytes isolated from wild type A/J mice were adoptively transferred to *H. hepaticus*-inoculated *RAG2* ^−/−^ recipients. Ninety days after adoptive transfers, all mice were sacrificed and ceca collected. Histologic evaluation revealed no significant differences in lesion scores between mice that received CD4^+^ lymphocytes from wild type, *ERα*-deficient, or *ERβ*-deficient donors (Fig. 4A). Analysis of the cecal expression of *CXCL9, IFN-γ, IL-4, IL-10, IL-12/23 p40, IL-17a, IL-17f and IL-23 p19* revealed no significant differences between the experimental and control mice (Fig. 4B), implying that estrogen signaling on a CD4^+^cell population is not responsible for the alterations in either mRNA cytokine expression or disease development demonstrated in the prior experiments. Furthermore, there were no significant correlations between lesion scores and cytokine expression (Table S4).

### Estrogen signaling on non-CD4^+^ lymphocytes suppresses disease severity

As the adoptive transfer of CD4^+^ cells with ablated ER expression did not result in modulation of disease, it was logical to speculate that estrogen signaling on a non-CD4^+^ cell population is responsible for the previously described disease modulation. To assess the role of ERα and ERβ on non-CD4^+^ lymphocyte cell populations, CD4^+^ lymphocytes from wild type donors were adoptive transferred into *H. hepaticus*-inoculated mice dually deficient for RAG2 and either ERα or ERβ expression. For a control group, wild type CD4^+^ lymphocytes were transferred into *H. hepaticus-*inoculated *RAG2* ^−/−^ mice with functional ERα and ERβ.

**Figure 5:**
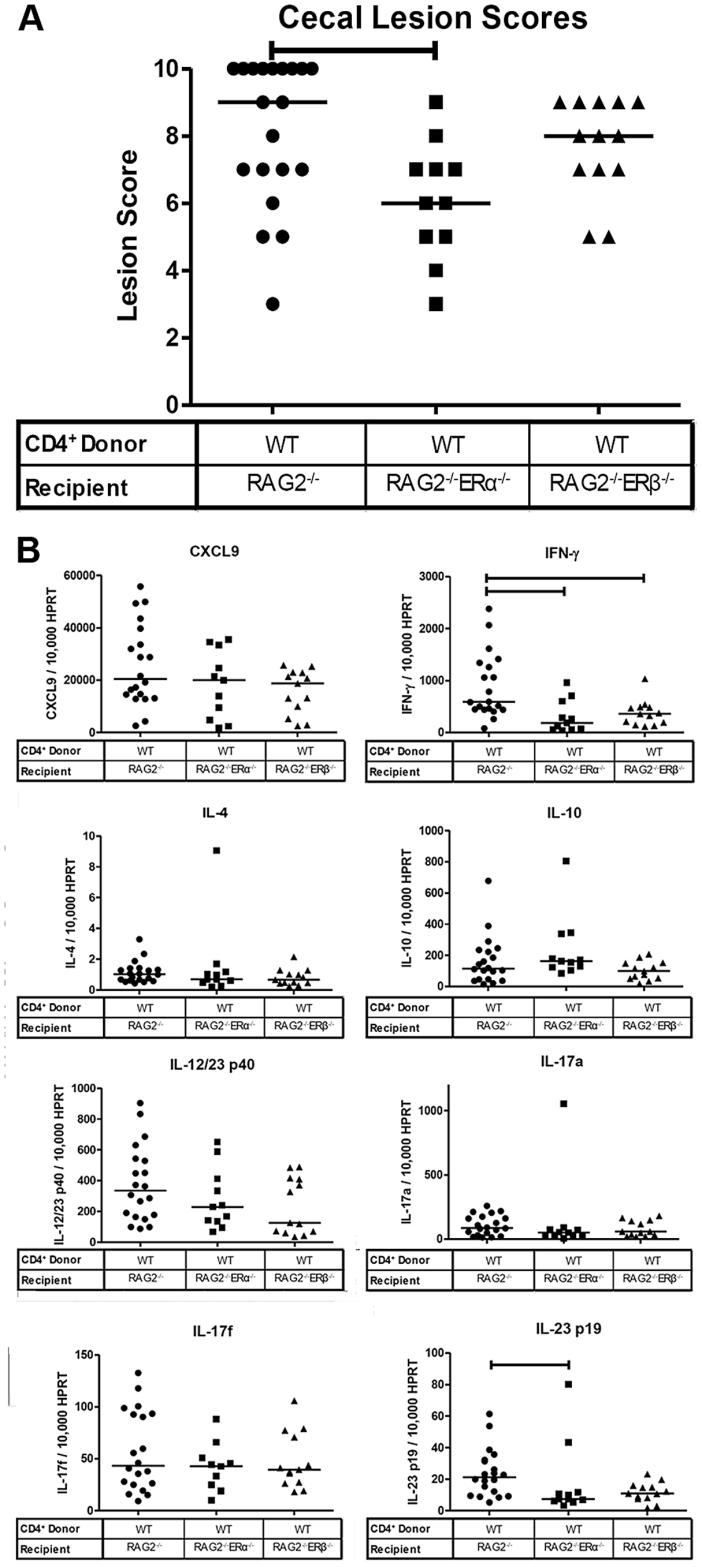
ERβ signaling on non-CD4^+^ cellular populations reduces cecal inflammation and IFN-γ expression. (A) Cecal lesion scores in female *H. hepaticus-*inoculated *ERα* ^−/−^ *RAG2* ^−/−^ (squares) or *ERβ* ^−/−^ *RAG2* ^−/−^ (triangles) female mice adoptively transferred wild type CD4^+^ lymphocytes (n = 11, 13 respectively). As a control group (circles), *RAG* ^−/−^ female mice were adoptively transferred wild type CD4^+^ lymphocytes (n = 20). Points represent the lesion scores of individual mice in each experimental group and horizontal lines represent median lesion scores. Lesion score data analyzed with an exact Kruskal-Wallis test followed by Dunn's post-hoc comparisons. (B) Real-time quantitative PCR measurements of cecal cytokine and chemokine mRNA expression levels in *RAG2* ^−/−^ mice deficient for either ERα or ERβ and adoptively transferred wild type CD4^+^ lymphocytes. All cytokine mRNA expression levels were normalized to *HPRT* expression. Data analyzed by an exact Kruskal-Wallis test followed by Dunn's post-hoc comparisons; each point represents the normalized expression of an individual mouse and horizontal lines represent medians. For all data analysis p≤0.05 considered significant and indicated by horizontal capped bars.

Adoptive transfer of CD4^+^ lymphocytes into mice lacking ERα resulted in significant decreases in disease severity (Fig. 5A). Associated with this decrease in disease severity were significant decreases in expression of *IFN-γ* and IL-23 p19. Moreover, expression of *IFN-γ*, *IL-23 p19* and *CXCL9*, expression showed positive correlations with lesion scores (Table S5). The reduction in disease development and associated cytokine gene expression changes in mice lacking ERα signaling on their non-CD4^+^ cells provides further support that estrogen signaling through ERβ can modulate mucosal inflammation.

## Discussion

The regulation of autoimmune diseases by GSHs is complex and multifaceted. There are many theories as to why women are more likely to develop an autoimmune disease. Many of these recognize the influence of GSHs; additionally most acknowledge that females, due to their evolutionary role to bear offspring, have an immune system programmed to accept exposure to allogeneic antigens, such as a developing fetus during pregnancy [5], [6]. Many diseases, such as MS, RA and SLE, demonstrate a female sex bias [4], [7]. In SLE, elevations in estrogen are associated with increased disease severity, while in MS and RA, increases in estrogen levels, such as occurs during pregnancy, have been shown to decrease disease severity [7], [37]. Additionally, animal models of MS [38]–[40] and RA [41] have recapitulated the beneficial effects of estrogen therapies.

The mucosal immune system of the gastrointestinal track also appears to be highly influenced by estrogen signaling. A female sex bias is seen in Crohn's disease, a subset of IBD [42], although it is small relative to the sex-bias of many other autoimmune-related diseases [4]. Furthermore, there is evidence that estrogen can modulate disease severity in IBD patients [15], [16], [43] and in animal models of IBD [17]–[20]. The influence of gonadal sex hormones is also apparent in our model as female *H. hepaticus-*infected A/J mice develop more severe intestinal inflammation than their male counterparts [29]. Additionally, there is a greater increase in the expression of inflammatory T_H_1 cytokines and chemokines in *H. hepaticus-*infected female mice, relative to male mice [29]. In light of these observations, we sought to investigate the effects of estrogen signaling on disease development and the associated cytokine responses.

In other animal models of autoimmune diseases such as experimental autoimmune encephalomyelitis (EAE), collagen-induced arthritis and DNB-induced colitis, high doses of estrogen effectively decrease disease severity [17], [21], [22]. Our findings that administration of a supraphysiologic estrogen dose decreases disease severity are consistent with these reports. This decrease in disease was associated with decreases in several cytokines including T_H_1-associated cytokines that typify *H. hepaticus* infection in A/J mice [24], [29]. However, this treatment also resulted in severe bone marrow depression and decreases in all but one of the cytokines evaluated, suggesting that this treatment was neither physiologically nor therapeutically relevant to understanding how estrogen can modulate mucosal inflammation. Nonetheless, these studies served as observation generating studies that stimulated the pursuit of additional studies centered on estrogen receptor signaling.

As both ERα and ERβ have been previously documented to be expressed on various cell types within the cecum [44]–[47], we sought to assess the role of specific ERs. To accomplish this, mice lacking either ERα or ERβ were inoculated with *H. hepaticus*. *H. hepaticus* infected mice with only functional ERβ signaling had decreased severity of disease that was associated with decreased *CXCL9* expression. Additionally there was a positive correlation between the expression of *CXCL9* and lesion scores. Interestingly, despite a lack of significant changes in the mRNA expression of *IL-12/23 p40* and *IFN-γ*, positive correlations were identified between both of these cytokines and lesion scores (Figure S2). From these results, it was reasonable to hypothesize that either 1) signaling through ERα is pro-inflammatory and removal of this signaling dampens disease or 2) preferential signaling through the remaining functional receptor in these mice, ERβ, results in dampening of disease.

We subsequently demonstrated decreases in both disease severity and cytokine gene expression when mice were treated with the ERβ agonist DPN. Other studies have also suggested a role for ERβ in modulating intestinal inflammation [11], in particular the publication by Looijer-van Langen et al. that demonstrates decreased ERβ expression in both human IBD patients, and in rodent models of IBD, including the IL-10^−/−^ mouse and the HLA-B27 rat [44]. The recent work by Saleiro et al. has further highlighted the protective properties of intestinal ERβ signaling by demonstrating the ability of ERβ signaling to inhibit the development of colitis-associated cancers in mice [23].

The finding that loss of ERα, which results in preferential signaling through ERβ, decreased disease severity, coupled with the finding that an ERβ agonist also decreased disease severity, suggests that signaling through ERβ abrogates the immune response and ultimately prevents development of inflammation. Interestingly, estrogen availability differed between these two groups (the ER knock-out mice had only endogenous estrogen signaling while the agonist treated mice were ovariectomized and supplemented with only one specific agonist), but both studies led to a similar conclusion that signaling through ERβ decreases disease severity. However, given these findings, we expected that ERβ-deficient mice would have increased disease severity, but this was not the case. The reasons for this are unknown, but we speculate that this model's maximum disease severity may have been reached and that neither depletion of ERβ nor endogenous estrogen signaling through ERα alone was sufficient to further exacerbate disease beyond this plateaued level.

Further experiments were performed to identify the cell types in which estrogen receptor signaling was modulating disease severity in this model. Previously published findings have shown intestinal epithelial cells express ERβ [8], [45] and CD4^+^ T cells express ERα [47] making it necessary to evaluate the involvement of both CD4^+^ and non-CD4^+^ cellular populations in disease modulation. To identify the cell type through which ER signaling modulates disease, an adoptive transfer model was utilized. When estrogen signaling on CD4^+^ T cells was ablated, no differences in histologic disease or mRNA expression were observed; additionally, no correlations between disease severity and mRNA expression were found (Table S4). However, when non-CD4^+^ cellular populations lacked ERα signaling, disease severity was decreased and *IFN-γ* and *IL-23 p19* expressions were lowered. Interestingly, there were also positive correlations between lesion scores and expression of *CXCL9, IFN-γ* and *IL-23 p19* (Table S5). Overall, these results suggest that the development of *H. hepaticus-*induced inflammation is independent of estrogen signaling on CD4^+^ T cells, and is dependent on estrogen signaling in a non-CD4^+^ cellular population.

Candidate non-CD4^+^ cells that express ERβ included epithelial cells and dendritic cells. Signaling through epithelial cell ERβ increases the development of tight junctions in the intestinal epithelium and decreases intestinal permeability [44]–[46], possibly resulting in decreased sampling of the luminal bacteria. Furthermore, treatment of colonic cells in culture with either 17β Estradiol or DPN, the ERβ agonist used in this study, results in increased epithelial resistance [44]. Increased epithelial resistance could decrease the ability of bacterial antigens to stimulate the immune system and could explain the decreased disease and associated expression of *IL-12/23 p40* and *IL-17a* seen in the ERβ agonist (DPN)-treated mice. Alternatively, as estrogen has been shown to influence the development and function of dendritic cells [48]–[50], ERβ signaling may be altering antigen presentation to the overall immune system, ultimately resulting in decreased disease development.

Two findings from these studies remain unexplained. First, our finding that signaling through specific estrogen receptors (e.g. ERβ) is associated with decreased disease is contradictory to the observation that, when inoculated with *H. hepaticus*, female mice develop more severe disease than males. The reasons for this paradox are unknown but likely involve complex interactions between other gonadal sex hormones such as progesterone in females and testosterone in males. Second, we also found unexplained perturbations in expression of some cytokines in mice given and ERα agonist or deficient in ERβ signaling. Because these were not associated with changes in disease severity, they were not pursued further and remain unexplained.

Collectively these studies, suggest that when estrogen signals solely through ERβ in a non-CD4^+^ cell population, an immunosuppressive effect occurs. This is supported by data from three distinct models: knock-out mice, ER agonist-treated mice, and an adoptive transfer model, all of which demonstrated decreased histologic disease and associated cytokine perturbations when estrogen signaling is limited to or supplemented with ERβ signaling. Moreover, this research is directly translatable to development of novel ERβ-targeting therapeutics that modulate mucosal inflammation in diseases such as IBD.

## Supporting Information

Table S1 **Spearman Correlation Coefficients for disease severity and cytokine mRNA expression in mice treated with estrogen.** Spearman's correlation coefficients were utilized to evaluate correlations between cytokine mRNA expression and disease severity in ovariectomized A/J mice sacrificed 90 days after *Helicobacter hepaticus* inoculation and implantation with either a subcutaneous pellet containing either 17β-estradiol (1.5 mg/pellet) or placebo. Corresponding p-values were adjusted by a false discovery rate (FDR) controlling method. For all analyses, p-values ≤.05 (after any adjustments) were regarded as significant and indicated by bold font.
(DOCX)

Table S2 **Spearman Correlation Coefficients for disease severity and cytokine mRNA expression in mice with altered ER signaling.** Spearman's correlation coefficients were utilized to evaluate correlations between cytokine mRNA expression and disease severity in ovariectomized ERα^−/−^, ERβ^−/−^, and heterozygous/wild type female A/J mice inoculated with *H. hepaticus* and necropsied 3 months post-inoculation. Corresponding p-values were adjusted by a false discovery rate (FDR) controlling method. For all analyses, p-values ≤.05 (after any adjustments) were regarded as significant and indicated by bold font.
(DOCX)

Table S3 **Spearman Correlation Coefficients for disease severity and cytokine mRNA expression in mice treated with ER agonist or placebo pellets.** Spearman's correlation coefficients were utilized to evaluate correlations between cytokine mRNA expression and disease severity in ovariectomized, female A/J mice administered a continuous-release pellet (placebo, ERα agonist (PPT) or ERβ agonist (DPN)), inoculated with *H. hepaticus* and necropsied three months post-inoculation. Corresponding p-values were adjusted by a false discovery rate (FDR) controlling method. For all analyses, p-values ≤.05 (after any adjustments) were regarded as significant and indicated by bold font.
(DOCX)

Table S4 **Spearman Correlation Coefficients for disease severity and cytokine mRNA expression in mice with altered ER signaling on CD4^+^ cell populations.** Correlations between cytokine mRNA expression and disease severity in *H. hepaticus-*inoculated *RAG2* ^−/−^ mice adoptively transferred *ERα* ^−/−^, *ERβ* ^−/−^, or wild type CD4^+^ lymphocytes were evaluated with Spearman's correlation coefficients. Corresponding p-values were adjusted by a false discovery rate (FDR) controlling method. For all analyses, p-values ≤.05 (after any adjustments) were regarded as significant. No significant correlations were found.
(DOCX)

Table S5 **Spearman Correlation Coefficients for disease severity and cytokine mRNA expression in mice with altered ER signaling on non-CD4^+^ cell populations.** Correlations between cytokine mRNA expression and disease severity in *H. hepaticus-*inoculated *ERα* ^−/−^ *RAG2* ^−/−^, *ERβ* ^−/−^ *RAG2* ^−/−^ or *RAG2* ^−/−^ mice adoptively wild type CD4^+^ lymphocytes were evaluated with Spearman's correlation coefficients. Corresponding p-values were adjusted by a false discovery rate (FDR) controlling method. For all analyses, p-values ≤.05 (after any adjustments) were regarded as significant and indicated by bold font.
(DOCX)
