# Supplementary material for: The Role of Estrogen Signaling in a Mouse Model of Inflammatory Bowel Disease: A Helicobacter Hepaticus Model
Source: PLoS One. 2014 Apr 7;9(4):e94209. doi: 10.1371/journal.pone.0094209 (PMC3978010; doi:10.1371/journal.pone.0094209)
Supplement: Table S1 — Spearman Correlation Coefficients for disease severity and cytokine mRNA expression in mice treated with estrogen. Spearman's correlation coefficients were utilized to evaluate correlations between cytokine mRNA expression and disease severity in ovariectomized A/J mice sacrificed 90 days after Helicobacter hepaticus inoculation and implantation with either a subcutaneous pellet containing either 17β-estradiol (1.5 mg/pellet) or placebo. Corresponding p-values were adjusted by a false discovery rate (FDR) controlling method. For all analyses, p-values ≤.05 (after any adjustments) were regarded as significant and indicated by bold font. (DOCX) [file pone.0094209.s001.docx]

|  |  | **Correlation Coefficient** | **Adjusted p-value** |
| --- | --- | --- | --- |
| **CXCL9** | **Cecal Lesion Score** | **0.68266** | **0.007133** |
| **IFN-γ** | **Cecal Lesion Score** | **0.70542** | **0.005669** |
| **IL-12/23 p40** | **Cecal Lesion Score** | **0.59164** | **0.026287** |
| **IL-10** | **Cecal Lesion Score** | **0.88746** | **0.000015** |
| IL-17a | Cecal Lesion Score | 0.38684 | 0.138819 |
| IL-17f | Cecal Lesion Score | 0.50972 | 0.054625 |
| IL-23 p19 | Cecal Lesion Score | 0.44601 | 0.092610 |
| IL-4 | Cecal Lesion Score | 0.52793 | 0.050801 |
