# Supplementary material for: The Role of Estrogen Signaling in a Mouse Model of Inflammatory Bowel Disease: A Helicobacter Hepaticus Model
Source: PLoS One. 2014 Apr 7;9(4):e94209. doi: 10.1371/journal.pone.0094209 (PMC3978010; doi:10.1371/journal.pone.0094209)
Supplement: Table S2 — Spearman Correlation Coefficients for disease severity and cytokine mRNA expression in mice with altered ER signaling. Spearman's correlation coefficients were utilized to evaluate correlations between cytokine mRNA expression and disease severity in ovariectomized ERα−/−, ERβ−/−, and heterozygous/wild type female A/J mice inoculated with H. hepaticus and necropsied 3 months post-inoculation. Corresponding p-values were adjusted by a false discovery rate (FDR) controlling method. For all analyses, p-values ≤.05 (after any adjustments) were regarded as significant and indicated by bold font. (DOCX) [file pone.0094209.s002.docx]

|  |  | **Correlation Coefficient** | **Adjusted p-value** |
| --- | --- | --- | --- |
| **CXCL9** | **Cecal Lesion Score** | **0.51459** | **0.001318** |
| **IFN-γ** | **Cecal Lesion Score** | **0.40081** | **0.019605** |
| **IL-12/23 p40** | **Cecal Lesion Score** | **0.35040** | **0.042024** |
| IL-10 | Cecal Lesion Score | 0.14510 | 0.449569 |
| IL-17a | Cecal Lesion Score | 0.29192 | 0.099215 |
| IL-17f | Cecal Lesion Score | 0.18303 | 0.406561 |
| IL-23 p19 | Cecal Lesion Score | 0.14975 | 0.449569 |
| IL-4 | Cecal Lesion Score | -0.06509 | 0.725972 |
