# Supplementary material for: The Role of Estrogen Signaling in a Mouse Model of Inflammatory Bowel Disease: A Helicobacter Hepaticus Model
Source: PLoS One. 2014 Apr 7;9(4):e94209. doi: 10.1371/journal.pone.0094209 (PMC3978010; doi:10.1371/journal.pone.0094209)
Supplement: Table S3 — Spearman Correlation Coefficients for disease severity and cytokine mRNA expression in mice treated with ER agonist or placebo pellets. Spearman's correlation coefficients were utilized to evaluate correlations between cytokine mRNA expression and disease severity in ovariectomized, female A/J mice administered a continuous-release pellet (placebo, ERα agonist (PPT) or ERβ agonist (DPN)), inoculated with H. hepaticus and necropsied three months post-inoculation. Corresponding p-values were adjusted by a false discovery rate (FDR) controlling method. For all analyses, p-values ≤.05 (after any adjustments) were regarded as significant and indicated by bold font. (DOCX) [file pone.0094209.s003.docx]

|  |  | **Correlation Coefficient** | **Adjusted p-value** |
| --- | --- | --- | --- |
| **CXCL9** | **Cecal Lesion Score** | **0.63908** | **0.002204** |
| **IFN-γ** | **Cecal Lesion Score** | **0.63977** | **0.002204** |
| IL-12/23 p40 | Cecal Lesion Score | 0.17505 | 0.850580 |
| **IL-10** | **Cecal Lesion Score** | **0.57106** | **0.007706** |
| IL-17a | Cecal Lesion Score | -0.06146 | 0.850580 |
| IL-17f | Cecal Lesion Score | -0.03729 | 0.856490 |
| IL-23 p19 | Cecal Lesion Score | -0.07941 | 0.850580 |
| IL-4 | Cecal Lesion Score | 0.09529 | 0.850580 |
