# Supplementary material for: The Role of Estrogen Signaling in a Mouse Model of Inflammatory Bowel Disease: A Helicobacter Hepaticus Model
Source: PLoS One. 2014 Apr 7;9(4):e94209. doi: 10.1371/journal.pone.0094209 (PMC3978010; doi:10.1371/journal.pone.0094209)
Supplement: Table S4 — Spearman Correlation Coefficients for disease severity and cytokine mRNA expression in mice with altered ER signaling on CD4+ cell populations. Correlations between cytokine mRNA expression and disease severity in H. hepaticus-inoculated RAG2 −/− mice adoptively transferred ERα −/−, ERβ −/−, or wild type CD4+ lymphocytes were evaluated with Spearman's correlation coefficients. Corresponding p-values were adjusted by a false discovery rate (FDR) controlling method. For all analyses, p-values ≤.05 (after any adjustments) were regarded as significant. No significant correlations were found. (DOCX) [file pone.0094209.s004.docx]

|  |  | **Correlation Coefficient** | **Adjusted p-value** |
| --- | --- | --- | --- |
| CXCL9 | Cecal Lesion Score | 0.244820 | 0.323180 |
| IFN-γ | Cecal Lesion Score | 0.210550 | 0.323180 |
| IL-12/23 p40 | Cecal Lesion Score | -0.059560 | 0.726214 |
| IL-10 | Cecal Lesion Score | -0.321070 | 0.266826 |
| IL-17a | Cecal Lesion Score | -0.158320 | 0.436657 |
| IL-17f | Cecal Lesion Score | -0.203840 | 0.323180 |
| IL-23 p19 | Cecal Lesion Score | 0.242790 | 0.323180 |
| IL-4 | Cecal Lesion Score | 0.083970 | 0.690271 |
