# Supplementary material for: The Role of Estrogen Signaling in a Mouse Model of Inflammatory Bowel Disease: A Helicobacter Hepaticus Model
Source: PLoS One. 2014 Apr 7;9(4):e94209. doi: 10.1371/journal.pone.0094209 (PMC3978010; doi:10.1371/journal.pone.0094209)
Supplement: Table S5 — Spearman Correlation Coefficients for disease severity and cytokine mRNA expression in mice with altered ER signaling on non-CD4+ cell populations. Correlations between cytokine mRNA expression and disease severity in H. hepaticus-inoculated ERα −/− RAG2 −/−, ERβ −/− RAG2 −/− or RAG2 −/− mice adoptively wild type CD4+ lymphocytes were evaluated with Spearman's correlation coefficients. Corresponding p-values were adjusted by a false discovery rate (FDR) controlling method. For all analyses, p-values ≤.05 (after any adjustments) were regarded as significant and indicated by bold font. (DOCX) [file pone.0094209.s005.docx]

|  |  | **Correlation Coefficient** | **Adjusted p-value** |
| --- | --- | --- | --- |
| **CXCL9** | **Cecal Lesion Score** | **0.38214** | **0.034906** |
| **IFN-γ** | **Cecal Lesion Score** | **0.51804** | **0.001581** |
| IL-12/23 p40 | Cecal Lesion Score | 0.23966 | 0.167339 |
| IL-10 | Cecal Lesion Score | -0.33963 | 0.060250 |
| IL-17a | Cecal Lesion Score | 0.06011 | 0.775893 |
| IL-17f | Cecal Lesion Score | -0.02991 | 0.847154 |
| **IL-23 p19** | **Cecal Lesion Score** | **0.56606** | **0.000621** |
| IL-4 | Cecal Lesion Score | 0.21934 | 0.190707 |
